# Treatment of Milk Sickness

**Published:** 1852-05

**Authors:** C. W. Davis

**Affiliations:** Carlisle


ART. VI.—*Treatment of Milk Sickness.*

*Messrs. Editors*—I WAS called to see a patient last fall, living in the region where sick stomach (as we term it) abounds, and found him laboring under obstinate constipation, and had been for some six days, vomiting almost continuedly. I pronounced the case Milk

Sickness, and directed an enema composed of sulph. magnesia, molasses and warm water to evacuate the rectum, and applied chloroform liniment over the region of the stomach, together with laudanum, poultices, &c., but to no purpose. The vomiting continued unabated. All medicines taken by the mouth were rejected immediately. Believing the vomiting to be caused by spasmodic contraction of the diaphragm and abdominal muscles, and there also being high nervous and vascular excitement, I bled the patient *ad syncope*, after which the vomiting ceased for some two hours, when I again opened the vein and took 12 oz. of blood, and administered sulphur and cream of tartar as a cathartic. The patient recovered in a few days.

Should you think the foregoing worthy, you may publish it, and in future I will give you a more detailed account of my practice in Milk Sickness. I am confident the foregoing treatment will prove successful in nine cases out of ten.

Yours truly, C. W. Davis, M. D.

Carlisle, April 12, 1852.

